# PCTAIRE Protein Kinase 1 (PCTK1) Suppresses Proliferation, Stemness, and Chemoresistance in Colorectal Cancer through the BMPR1B-Smad1/5/8 Signaling Pathway

**DOI:** 10.3390/ijms241210008

**Published:** 2023-06-11

**Authors:** Po-Li Wei, Chien-Yu Huang, Tung-Cheng Chang, Jang-Chun Lin, Cheng-Chin Lee, G. M. Shazzad Hossain Prince, Precious Takondwa Makondi, Angelina Wong-Ying Chui, Yu-Jia Chang

**Affiliations:** 1 Division of Colorectal Surgery, Department of Surgery, Taipei Medical University Hospital, Taipei Medical University, Taipei 11031, Taiwan; poliwei@tmu.edu.tw; 2 Department of Surgery, College of Medicine, School of Medicine, Taipei Medical University, Taipei 11031, Taiwanprincegmsh@tmu.edu.tw (G.M.S.H.P.); 3 Cancer Research Center and Translational Laboratory, Department of Medical Research, Taipei Medical University Hospital, Taipei Medical University, Taipei 11031, Taiwan; 4 Graduate Institute of Cancer Biology and Drug Discovery, Taipei Medical University, Taipei 11031, Taiwan; 5 School of Medicine, National Tsing Hua University, Hsinchu 30013, Taiwan; cyhuang@life.nthu.edu.tw; 6 Institute of Molecular and Cellular Biology, National Tsing Hua University, Hsinchu 30013, Taiwan; 7 Department of Pathology, Wan Fang Hospital, Taipei Medical University, Taipei 11696, Taiwan; 8 Division of Colon and Rectal, Department of Surgery, Shuang Ho Hospital, Taipei Medical University, Taipei 11031, Taiwan; 9 Department of Radiotherapy and Oncology, Shuang Ho Hospital, Taipei Medical University, Taipei 11031, Taiwan; k2220707@gmail.com; 10 Graduate Institute of Medical Sciences, College of Medicine, Taipei Medical University, Taipei 11031, Taiwan; kerwinpipi@gmail.com; 11 Kamuzu Central Hospital, National Cancer Center, Lilongwe P.O. Box 149, Malawi; khondipule@gmail.com; 12 Department of Radiation Oncology, Taipei Medical University Hospital, Taipei 11031, Taiwan; amobi6@hotmail.com; 13 Graduate Institute of Clinical Medicines, College of Medicine, Taipei Medical University, Taipei 11031, Taiwan; 14 Cell Physiology and Molecular Image Research Center, Wan Fang Hospital, Taipei Medical University, Taipei 11031, Taiwan

**Keywords:** PCTK1, CRC, Smad, BMPR1B, chemoresistance, stemness

## Abstract

Colorectal cancer (CRC) is the third most common cancer and a leading cause of cancer-related mortality worldwide. Even with advances in therapy, CRC mortality remains high. Therefore, there is an urgent need to develop effective therapeutics for CRC. PCTAIRE protein kinase 1 (PCTK1) is an atypical member of the cyclin-dependent kinase (CDK) family, and the function of PCTK1 in CRC is poorly understood. In this study, we found that patients with elevated PCTK1 levels had a better overall survival rate in CRC based on the TCGA dataset. Functional analysis also showed that PCTK1 suppressed cancer stemness and cell proliferation by using PCTK1 knockdown (PCTK1-KD) or knockout (PCTK1-KO) and PCTK1 overexpression (PCTK1-over) CRC cell lines. Furthermore, overexpression of PCTK1 decreased xenograft tumor growth and knockout of PCTK1 significantly increased in vivo tumor growth. Moreover, knockout of PCTK1 was observed to increase the resistance of CRC cells to both irinotecan (CPT-11) alone and in combination with 5-fluorouracil (5-FU). Additionally, the fold change of the anti-apoptotic molecules (Bcl-2 and Bcl-xL) and the proapoptotic molecules (Bax, c-PARP, p53, and c-caspase3) was reflected in the chemoresistance of PCTK1-KO CRC cells. PCTK1 signaling in the regulation of cancer progression and chemoresponse was analyzed using RNA sequencing and gene set enrichment analysis (GSEA). Furthermore, PCTK1 and Bone Morphogenetic Protein Receptor Type 1B (BMPR1B) in CRC tumors were negatively correlated in CRC patients from the Timer2.0 and cBioPortal database. We also found that BMPR1B was negatively correlated with PCTK1 in CRC cells, and BMPR1B expression was upregulated in PCTK1-KO cells and xenograft tumor tissues. Finally, BMPR1B-KD partially reversed cell proliferation, cancer stemness, and chemoresistance in PCTK1-KO cells. Moreover, the nuclear translocation of Smad1/5/8, a downstream molecule of BMPR1B, was increased in PCTK1-KO cells. Pharmacological inhibition of Smad1/5/8 also suppressed the malignant progression of CRC. Taken together, our results indicated that PCTK1 suppresses proliferation and cancer stemness and increases the chemoresponse of CRC through the BMPR1B–Smad1/5/8 signaling pathway.

## 1. Introduction

Colorectal cancer (CRC) is the third most common cancer worldwide and the second leading cause of cancer-related death [1]. Despite advances in patient screening procedures and substantial progress in therapeutic approaches, the mortality rate of CRC remains high. Chemotherapy is the standard treatment for metastatic CRC (mCRC). The cytotoxic chemotherapeutic agents 5-fluorouracil (5-FU), capecitabine (a prodrug of 5-FU), oxaliplatin, and irinotecan (IRI), used in combination with an epidermal growth factor receptor or a vascular endothelial growth factor antagonist, constitute the primary treatment for mCRC. However, treatment failure due to therapeutic resistance leads to a poor prognosis [2]. Therefore, identifying a therapeutic target and regulatory mechanism relevant to prognosis prediction and therapeutic response in mCRC may considerably improve patients’ clinical outcomes.

PCTAIRE protein kinase 1 (PCTK1), PCTK2, and PCTK3 are highly conserved serine/threonine kinases that belong to the cyclin-dependent kinase (CDK) family of protein kinases [3]. Also known as PCTAIRE1 and CDK16 (cyclin-dependent kinase 16), PCTK1 is expressed throughout the body and is highly expressed in terminally differentiated tissues, including the brain and testes [4,5,6]. PCTK1 has a central kinase domain with high similarity to conventional CDKs, and this region is flanked by unique N- and C-terminal domains [7]. The N-terminal domain is vital because its deletion leads to the loss of kinase activity in vitro [8]. However, the mechanisms underlying PCTK1 activation remain unclear. PCTK1 also plays an integral role in cell proliferation and anti-apoptosis [9,10,11,12]; the late G_2_ mitotic phase is arrested in PCTK1 knockdown (KD) cancer cells [9]. Moreover, PCTK1 is overexpressed in multiple types of cancer cells, including CRC and melanoma, breast, and prostate cancer cells [13]. Therefore, PCTK1 may be a potential target for therapeutic intervention in cancers.

Bone morphogenetic proteins (BMPs) include 15 mammalian members belonging to the transforming growth factor-β (TGFβ) superfamily [14]. BMP ligands initiate the signal transduction cascade by binding to type 1 receptors (BMPR1A or BMPR1B) and type II receptors (BMPRII) to form a heterotetrameric complex. The constitutively active type II receptor then transphosphorylates the type I receptor, and the type I receptor phosphorylates the receptor-regulated Smads (Smad1/5/8). Phosphorylated Smad1/5/8 associates with co-Smad (Smad4). This involves the complex translocation into the nucleus and association with coactivators or corepressors to regulate gene expression [15]. This suggests that BMPs play an essential role in tumor cell initiation and proliferation. A BMPR1A mutation was noted in juvenile polyposis syndrome [16]. In the majority of sporadic CRC cases, the BMP-Smad pathway is inactivated [17]. Furthermore, Smad7, BMP2, and BMP4 were shown to be associated with CRC in genome-wide association studies [18]. However, the roles of PCTK1 and the Smad–BMPR1B signaling pathway remain unclear. We investigated the relationships among CRC progression, chemoresistance, and PCTK1 expression. PCTK1 negatively regulated CRC progression and chemoresistance through the Smad–BMPR1B signaling pathway.

## 2. Results

### 2.1. High PCTK1 Expression Was Associated with a More Favorable CRC Prognosis

**Figure 1:**
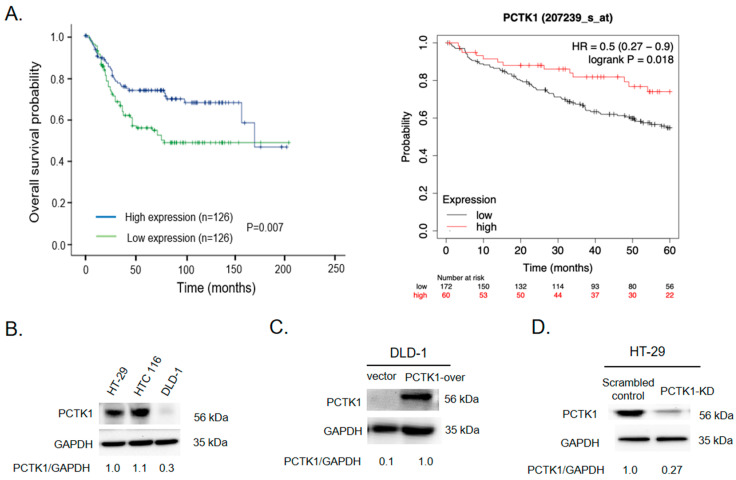

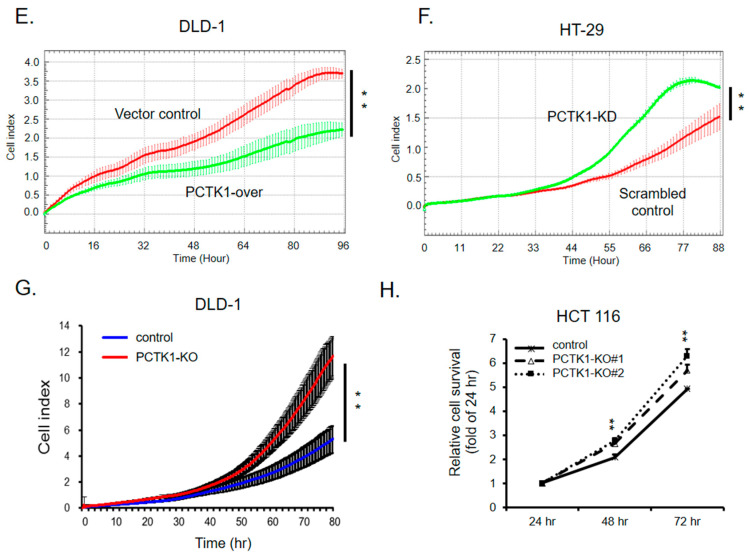
Effect of PCTK1 on CRC progression. (**A**) Patients with CRC were stratified by PCTK1 expression and subjected to Kaplan–Meier analysis of overall survival in GEO datasets (left panel: GSE41258, *n* = 252; right panel: GSE17538, *n* = 232). (**B**) PCTK1 protein expression levels were determined through Western blotting in three CRC cell lines. (**C**,**D**) Overexpression and knockdown stable cell lines were generated. (**E**–**H**) Cell proliferation was evaluated using the biosensor and SRB assay. Values are presented as means ± standard errors of the mean., ** *p* < 0.01.

First, to explore the role of PCTK1 in CRC, we analyzed the correlation between PCTK1 expression and clinical outcomes in CRC by using public databases. As shown in Figure 1A (left panel), lower expression of PCTK1 in a CRC specimen was significantly associated with poor prognosis in GSE41258 datasets (*n* = 252, *p* = 0.007). Likewise, in Figure 1A (right panel), the analysis of another CRC dataset (GSE17538, *n* = 232, *p* = 0.018, HR = 0.5 (0.27–0.9)) also indicated that lower PCTK1 leads to poor overall survival. Those patients with higher PCTK1 expression had significantly more favorable survival outcomes, suggesting that PCTK1 acts as a suppressor of CRC progression. To further investigate the mechanism of PCTK1 in regulating CRC progression, we tried to manipulate the PCTK1 gene and generated stable cell lines with stably overexpressing PCTK1 or knockdown of PCTK1 in CRC cells. The expression of PCTK1 in different human colon adenocarcinoma cell lines (HT-29, DLD-1, and HCT116) was determined through Western blotting. As shown in Figure 1B, PCTK1 was expressed in all the CRC cell lines but was expressed in a very low amount in DLD-1 cells. So, based on the evaluation of the expression levels of PCTK1 among different CRC cell lines, we chose the cell lines for the following experiments. Next, stable KD of PCTK1 (PCTK-KD) and a control cell line (scrambled control) were established in HT-29 cells. Control and stably overexpressing PCTK1 cells were also generated (vector and PCTK1-over) in DLD-1 cells. The expression of PCTK1 in stably overexpressing and KD cell lines was confirmed using Western blotting (Figure 1C,D). Furthermore, two CRC cell lines, DLD-1 and HCT116, were employed to create PCTK1-KO cell lines by the removal of exons 2 through 6 by using CRISPR/Cas9 technology (Figure S1).

### 2.2. PCTK1 Expression Suppressed CRC Cell Proliferation

Next, we examined whether dysregulation of PCTK1 expression affected cell proliferation in CRC. The cell index curves, constructed from the RTCA results, revealed dramatic differences between PCTK1-over, PCTK1-KD, and PCTK1-KO cells and their controls. Overexpression of PCTK1 in DLD-1 cells resulted in decreased cell proliferation. However, PCTK1-KD or PCTK1-KO cells resulted in a higher proliferation rate relative to the control cells (Figure 1E–H). These results from gain-of-function and loss-of-function experiments indicated that PCTK1 suppressed the proliferation of CRC cells.

### 2.3. PCTK1 Inhibited Tumorigenesis and Tumor Growth In Vivo

**Figure 2:**
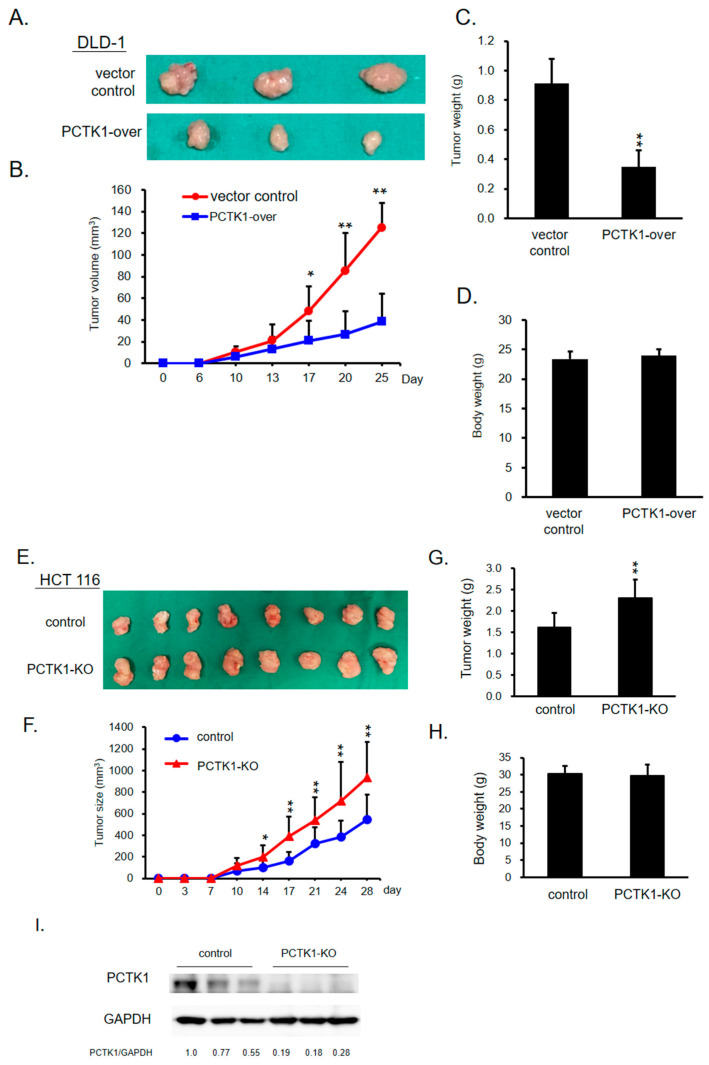
Effect of PCTK1 on tumor cell proliferation in a xenograft model. (**A**,**E**) Representative images of xenograft tumors isolated from mice, implanted with control cells (DLD-1 or HCT 116 cells), DLD-1 PCTK1-over, or HCT 116 PCTK1-KO cells. DLD-1 control and PCTK1-over xenografts contained 6 mice/group, and HCT 116 control and PCTK1-KO xenografts contained 8 mice/group. (**B**,**F**) Tumor volume and body weight (**D**,**H**) were measured twice a week, and the mass of the xenografts were determined after the mice were killed (**C**,**G**). (**I**) PCTK1 expression of HCT 116 xenograft tumors was determined through Western blotting. Data are presented as means ± standard deviations. * *p* < 0.05, ** *p* < 0.01.

PCTK1-over versus control DLD-1 cells and PCTK1-KO versus control HCT116 cells were subcutaneously implanted in nude mice, and the effect on tumor initiation and progression was monitored. Xenograft tumor growth, volume, and weight (Figure 2A–C) in the mice implanted with PCTK1-over cells were substantially lower than those in the control mice. Xenograft tumor growth, volume, and weight were consistently significantly higher (Figure 2E–G) in the PCTK1-KO group than in the control group. The expression of PCTK1 in mouse tumor tissues was assessed through Western blotting (Figure 2I). Taken together, these results suggested that PCTK1 contributes to tumor suppression in CRC in vivo.

### 2.4. PCTK1 Negatively Regulated Chemoresistance in CRC

**Figure 3:**
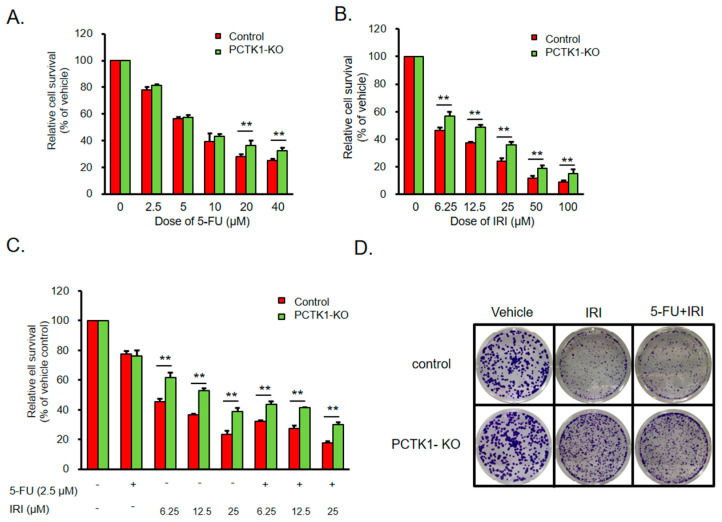

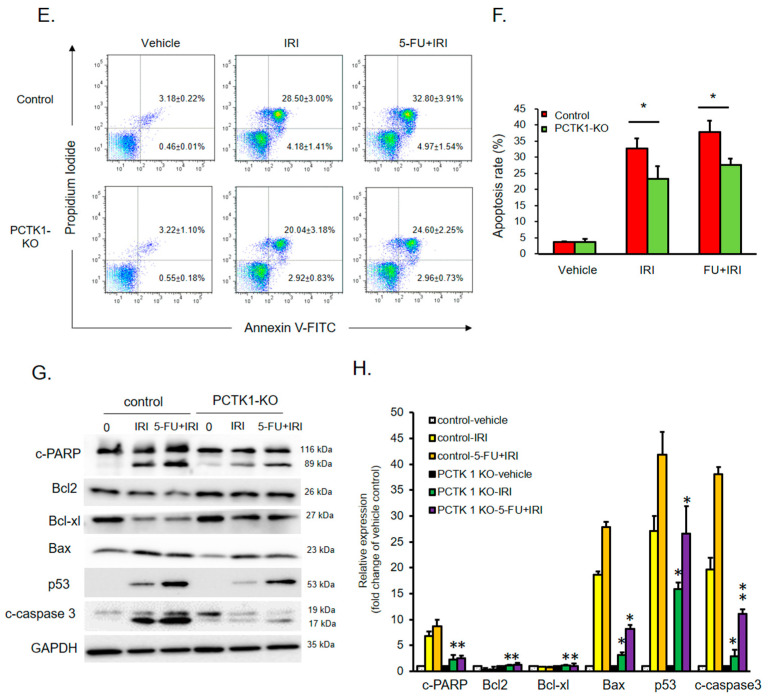
PCTK1 knockout made CRC cells chemoresistant. (**A**–**C**) Viability of HCT 116 control and PCTK1-KO cells was measured using the SRB assay in the presence of the chemotherapeutics fluorouracil (5-FU; 0–40 μM) and irinotecan (IRI; 0–100 μM). The vehicle control was set to 100%, and the ratio OD_515 nm_ values were used to represent the viability ratio. An asterisk represents the significance between HCT 116 control and PCTK1-KO cells. (**D**) Illustrations of the colony formation experiment using control and PCTK1-KO HCT 116 cells that were given IRI alone or IRI-5-FU treatment. (**E**,**F**) Annexin V/FITC and propidium iodide double labeling was used to identify cell apoptosis after a 48 h chemotherapy treatment in HCT 116 control and PCTK1-KO cells. (**G**,**H**) Western blotting was used to compare the apoptosis-related molecule expression in HCT 116 control and PCTK1-KO cells treated with IRI alone or IRI-5-FU. (**F**,**H**) E and G’s quantifications are presented in bar graphs. The means and standard deviations of the data are displayed. * *p* < 0.05, ** *p* < 0.01.

Chemotherapy is the mainstream therapeutic approach for CRC treatment [19]. However, chemoresistance is a major obstacle in the treatment of cancers and causes cancer recurrence [20]. We found that CRC patients with lower PCTK1 level had poor overall survival (Figure 1A). Therefore, we were curious to investigate whether PCTK1 mediates chemoresponse in CRC. The HCT 116 control and PCTK1-KO cells were treated with various doses of 5-fluouracil (5-FU) and irinotecan (IRI), one of the standard first-line chemotherapeutics for CRC. The data showed that the cell viability decreased in a dose-dependent manner. However, a significance difference in cell viability between the groups was only noted under the highest dose of 5-FU (Figure 3A). Several clinical studies have reported that IRI significantly reduces therapeutic resistance to 5-FU-based therapy for CRC [21,22,23]. Combination chemotherapy regimens that have been shown to achieve better therapeutic outcome are often preferred over single agents for mCRC treatment. Herein, the viability of HCT 116 PCTK1-KO cells under various doses of IRI treatments were significantly higher compared to the control cells (Figure 3B). Moreover, HCT 116 PCTK1-KO increased cell viability (relative to control cells) in response to both IRI alone and in combination with a low dose of 5-FU (Figure 3C). Similarly, the colony formation ability even under both IRI treatment alone and in combination with 5-FU was higher in HCT 116 PCTK1 knockout cells than the control cells (Figure 3D). To further determine the effects of PCTK1 on the chemotherapeutic sensitivity of CRC, an annexin V/propidium iodide double staining assay was used to evaluate the cell apoptosis rate of HCT 116 control versus HCT 116 PCTK1-KO cells in different chemotherapy regimens. Apoptosis was reduced in the HCT 116 PCTK1-KO cells under both IRI single treatment (32.68% ± 3.17% versus 23.32% ± 3.98%) and IRI–5-FU combination regimens (37.77% ± 3.53% versus 27.56% ± 1.99%; Figure 3E,F). The expression of apoptosis-related proteins in the HCT 116 control versus PCTK1-KO cells was evaluated through Western blotting following chemotherapeutic drug treatment. Following IRI treatment and IRI–5-FU treatment, the reduction in the expression of antiapoptotic Bcl-2 relative Bcl-xL and an increase in the expression of apoptotic proteins Bax, c-PARP, c-caspase3, and p53 were less in PCTK1-KO cells than those in control cells (Figure 3G,H). These data indicated that knockout of PCTK1 suppressed the chemoresponse by regulating caspase-dependent apoptosis. These results also suggested that PCTK1 is a predictive biomarker for the sensitivity of IRI treatment (either alone or combined with 5-FU) in CRC treatment regimen. Additionally, using the ROC Plotter platform, we also evaluated the response of colorectal cancer patients to chemotherapy treatment according to their PCTK1 expression levels. Consistent with our findings, patients exhibiting poor responsiveness to chemotherapy (*p* = 0.0068) had comparatively lower PCTK1 expression (Figure S2).

### 2.5. PCTK1 Expression Altered the Cancer Stem Cell Characteristics of CRC Cells

**Figure 4:**
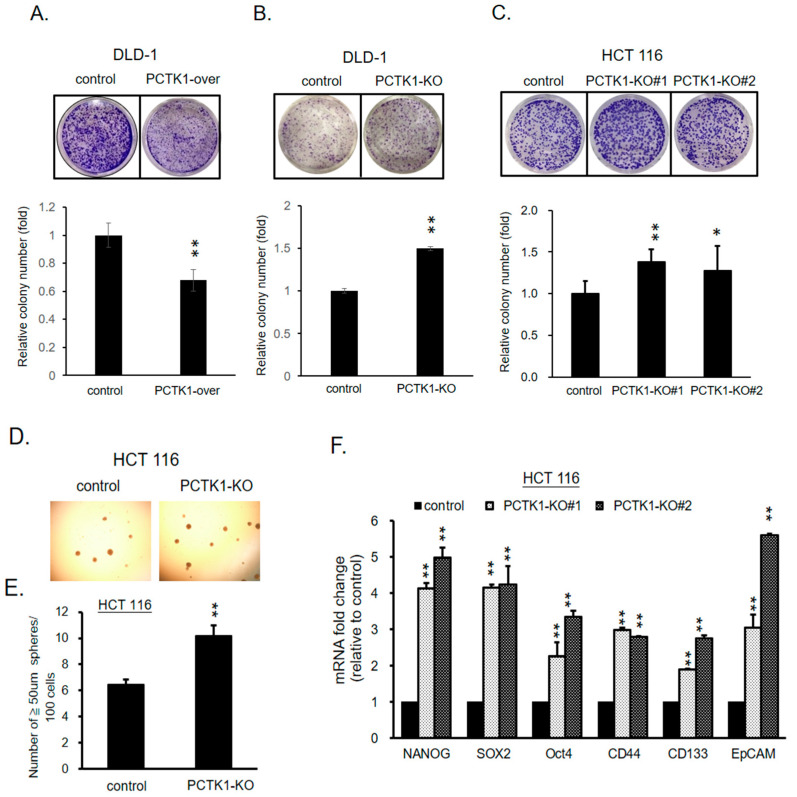
PCTK1 suppressed CSC-associated phenotypes of CRC cells. The clonogenic assay and sphere formation assay were employed to measure the stemness of CRC cells. (**A**–**D**). (**A**–**C**) Representative images of colony formation in control, PCTK1-over, and PCTK1-KO cells. Colonies were quantified by ImageJ and presented as a fold change of control. (**D**) Representative images of the sphere-forming ability of HCT 116 control and PCTK1-KO cells; statistical analysis of the number of spheres (**E**). (**F**) mRNA levels of NANOG, SOX2, Oct-4, CD44, CD133, and EpCAM in the control and PCTK1-KO cells were determined through qPCR. Values are presented means ± standard errors of the mean from at least three independent experiments and the statistical significance are determined through the two-tailed Student’s *t* test. * *p* < 0.05, ** *p* < 0.01 relative to control cells.

Cancer stem cells (CSCs) are a subpopulation of tumor cells that can drive tumor initiation and are responsible for maintaining tumor heterogeneity, enhanced proliferation, and therapeutic resistance [24]. Our data indicated that PCTK1 suppressed tumor growth and chemoresponse in CRC. To evaluate the effect of PCTK1 on the CSC characteristics of CRC cells, a clonogenic assay was performed. PCTK1 overexpression blocked colony formation in DLD-1 cells significantly, and PCTK1-KO DLD-1 and HCT116 resulted in a greater colony formation ability relative to controls (Figure 4A–C). A study reported that spheroid formation is an index of cells’ self-renewal capacity, which is the defining characteristic of CSCs [25]. The effect of PCTK1 on the sphere-forming ability of CRC cells was examined through a sphere formation assay in which the control and PCTK1-KO HCT116 cells were cultured in serum-free medium containing B27, bFGF, and EGF. Under stem cell growth conditions, the PCTK1-KO cells formed more spheres than the control cells (Figure 4D,E). A review paper noted that several surface markers and pluripotency transcription factors were implicated to identify CSCs in CRC [26]. Herein, PCTK1-KO cells consistently showed an upregulation in the expression of CSC markers (NANOG, SOX2, Oct4, CD44, CD133, and EpCAM) (Figure 4F). In contrast, overexpression of PCKT1 led to significant downregulation of all these CSC markers (Figure S4).

### 2.6. PCTK1 Suppressed Cell Proliferation, CSC Properties, and Chemoresponse through BMPR1B–Smad Signaling

**Figure 5:**
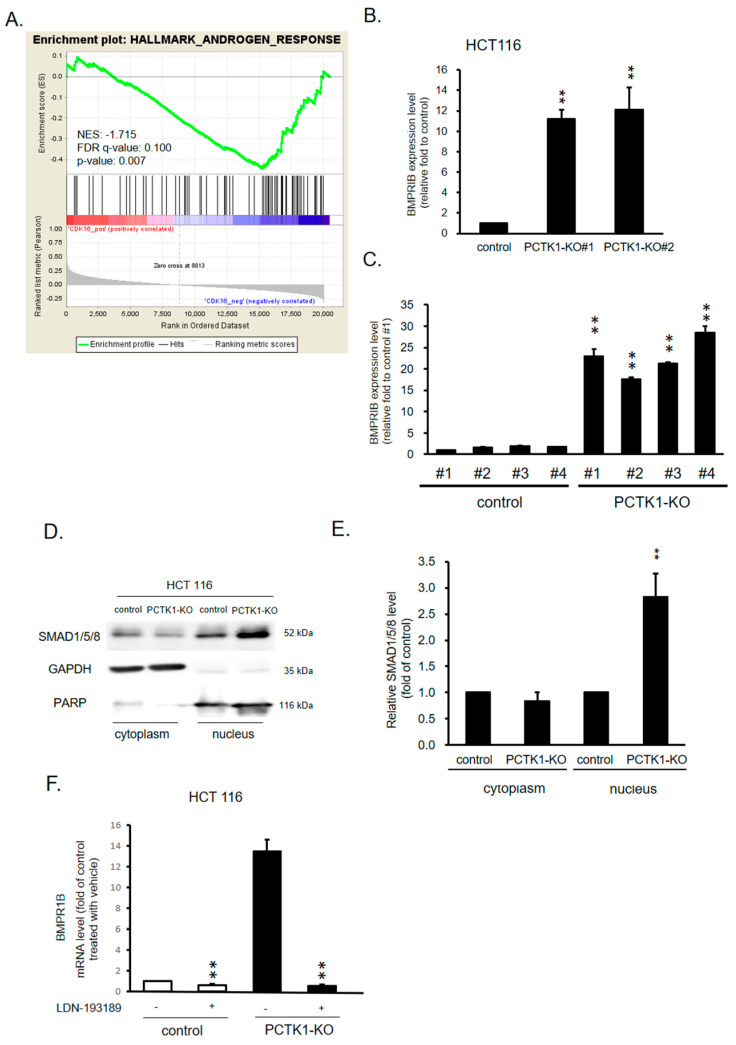
PCTK1 suppressed BMPR1B expression through Smad1/5/8 in CRC cells. (**A**) RNA sequencing-based GSEA indicated the enrichment of genes in regulating an androgen response gene set. Genes were ranked according to log2 fold changes in gene expressions. The normalized enrichment score, FDR q-value, and p value are indicated. (**B**,**C**) The expression of BMPR1B in HCT 116 PCTK1-KO cells and xenograft tumors was determined through qPCR. (**D**) Smad1/5/8 expression in cell fractions of HCT 116 control and PCTK-KO cells was determined by Western blot analysis and then quantified (**E**). (**F**) BMPR1B expression was determined through qPCR in HCT 116 control and PCTK1-KO cells treated with Smad1/5/8 inhibitors, (LDN193189 and palovarotene). Data are presented as means ± standard deviations. ** *p* < 0.01.

To investigate the direct gene targets and signaling pathways regulated by PCTK1 in cell proliferation and the therapeutic response in CRC, GSEA analysis was performed on the RNA sequencing data in PCTK1-overexpressing and control CRC cells. PCTK1 overexpression was negatively correlated with the androgen response signaling pathway, and based on GSEA, a considerable reduction in BMPR1B expression was observed in the androgen response gene sets (Figure 5A). To validate these results, RT-PCR was performed. BMPR1B expression was upregulated in the HCT 116 PCTK1-KO cells and the xenograft tumor tissues (Figure 5B,C). A previous study indicated that BMPR1B is involved in the TGFβ/Smad signaling pathway and is associated with CRC risk [27]. BMPR1B-activated receptor activates SMAD proteins (R-SMADs), Smad1/5/8, then complexes with Smad4 and triggers its nuclear translocation. On the other hand, Smad4 can also regulate the canonical BMP/Smad signaling via a positive feedback mechanism [28,29] Thus, we evaluated the nuclear translocation of Smad1/5/8 in HCT 116 PCTK1-KO cells using Western blotting. The expression of Smad1/5/8 in the nucleus was upregulated in the HCT 116 PCTK1-KO cells (Figure 5D,E). GAPDH and PARP were used as the cytoplasmic and nuclear internal controls, respectively. The phosphorylated Smad1/5/8–Smad4 complex has been reported to transmit signals to the nucleus and then activate BMPR1B transcription [28,29]. To further clarify the role of Smad1/5/8 in the signaling pathway of the PCTK1–BMPR1B axis, BMPR1B expression was determined in HCT 116 control and PCTK1-KO cells treated with the Smad1/5/8 inhibitors LDN193189 and palovarotene. In all cells, LDN193189 and palovarotene suppressed BMPR1B expression, which confirmed the positive feedback mechanism of Smad1/5/8 in the canonical BMP/Smad signaling axis (Figure 5F). Together, these results suggested that PCTK1 regulates BMPR1B via a positive feedback mechanism of Smad1/5/8.

### 2.7. BMPR1B Knockdown Partially Inhibited the Promotion of PCTK1 Knockout on CRC Cell Malignant Phenotype and Chemoresisitance

**Figure 6:**
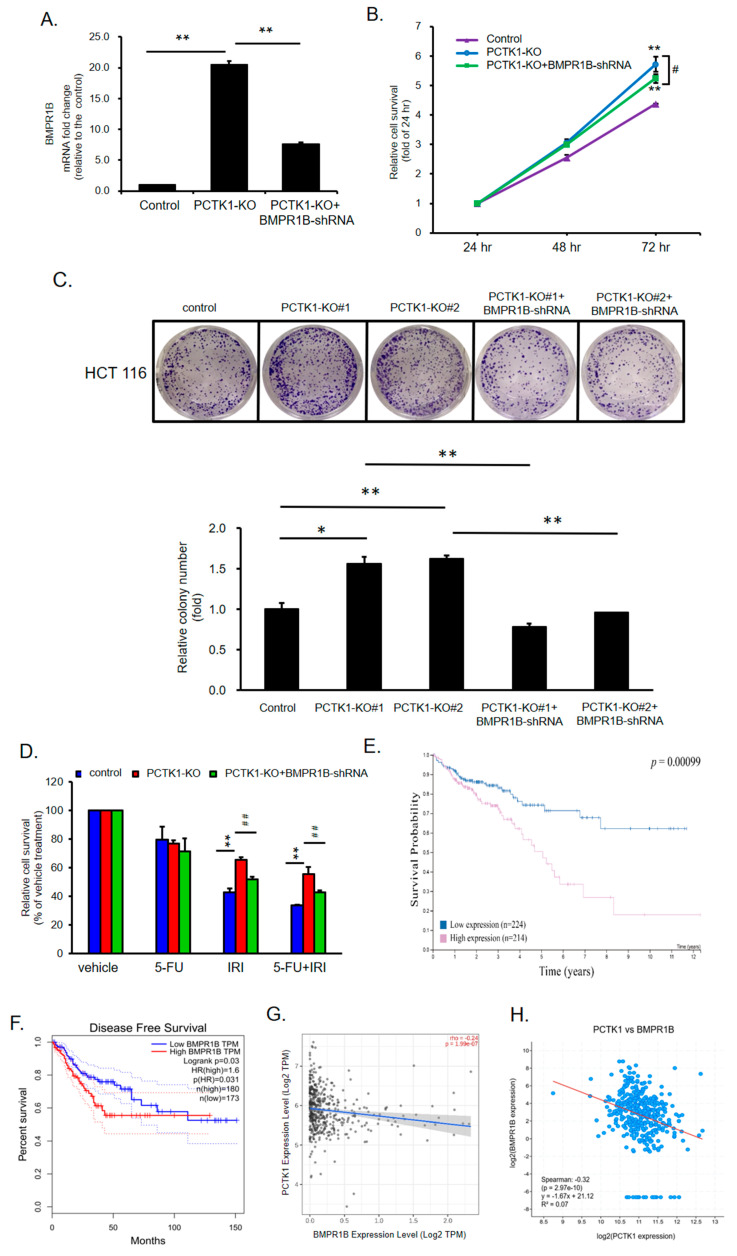
PCTK1 suppressed CRC malignancy through BMPR1B downregulation. (**A**) BMPR1B-KD cells were generated by transfecting BMPR1B-shRNA into PCTK1-KO HCT116 cells. BMPR1B expression in control, PCTK1-KO, and PCTK1-KO with BMPR1B-shRNA was determined through qPCR. (**B**) Cell proliferation was detected using the SRB assay. (**C**) Representative image of colony formation (stained with 1% crystal violet). Colonies were quantified by ImageJ and presented as mean ± SD fold of control. (**D**) Cell viability was measured using the SRB assay following 48 h chemotherapy treatment. Kaplan–Meier survival curves comparing the high and low expression of BMPR1B in CRC cells. (**E**) Overall survival analysis conducted using the Human Protein Atlas. (**F**) Disease-free survival analysis of TCGA data was performed using the GEPIA. (**G**,**H**) Correlation between PCTK1 and BMPR1B in patients with CRC was analyzed using the Timer 2.0 database and cBioPortal. * *p* < 0.05, ** *p* < 0.01, ^#^ *p* < 0.05, ^##^ *p* < 0.01.

To determine whether PCTK1-mediated cell proliferation, CSC properties, and chemoresponse are mediated by BMPR1B, BMPR1B was knocked down in PCTK1-KO cells by using shRNA-based transfection. The data revealed that the knockdown of BMPR1B partially reversed the proliferation activity and colony formation ability of the HCT 116 PCTK1-KO cells (Figure 6B,C). Moreover, compared with the HCT 116 PCTK1-KO/control-shRNA cells, cell viability was reduced in the HCT 116 PCTK1-KO/BMPR1B-shRNA cells in response to both IRI and IRI–5-FU treatment. However, the cell viability of HCT 116 PCTK1-KO/BMPR1B-shRNA cells was higher than that of the control cells (Figure 6D). The data suggested PCTK1 suppressed cell proliferation, CSC properties, and chemoresistance through the inhibition of BMPR1B. To verify the role of BMPR1B in CRC, we further examined the role of BMPR1B in the clinical outcomes of CRC from the Human Protein Atlas and the GEPIA dataset. The data showed high BMPR1B expression was associated with poor overall survival and disease-free survival in CRC (Figure 6E,F). Moreover, there was negative co-expression of PCTK1 with BMPR1B in CRC patients using the Timer 2.0 dataset (rho = −0.24, *p* = 1.99 × 10^−7^) and cBioPortal (Spearman’s correlation coefficient −0.32, *p* = 2.97 × 10^−10^) (Figure 6G,H).

Additionally, we were curious to learn if the negatively associated PCTK1 co-expressed gene sets and positively associated BMPR1B gene sets in CRC share any common pathway. So, to further investigate the roles of PCTK1 and BMPR1B in CRC, we retrieved a list of co-expression genes from the Colorectal Adenocarcinoma (TCGA, Firehose Legacy) dataset available in cBioPortal. Because of the negative correlation between PCTK1 and BMPR1B, we identified 131 negative co-expression genes with PCTK1 and 956 positive co-expression genes with BMPR1B. By intersecting co-expression gene lists, we obtained 69 candidate co-expression genes (Figure S3A). Subsequently, we performed GO/KEGG enrichment analysis on PCTK1 and BMPR1B together with candidate co-expressed genes, which showed seven significantly enriched pathways. GO/KEGG results included CC: caveola and MF: cytokine binding, while the enriched KEGG pathways included Fluid shear stress and atherosclerosis, Complement and coagulation cascades, Pertussis, Cell adhesion molecules, and Staphylococcus aureus infection (Figure S3B). Notably, pathways related to cancer, such as MF: cytokine binding and KEGG: Cell adhesion molecules, suggested that the downregulation of PCTK1 and upregulation of BMPR1B may influence cancer cell proliferation and drug sensitivity through mechanisms associated with cell adhesion molecules.

### 2.8. Pharmacological Targeting BMPR1B-SMAD1/5/8 Signaling with Small Molecules Inhibited the Promotion of PCTK1 Knockout on CRC Cell Malignant Phenotype and Chemoresisitance

**Figure 7:**
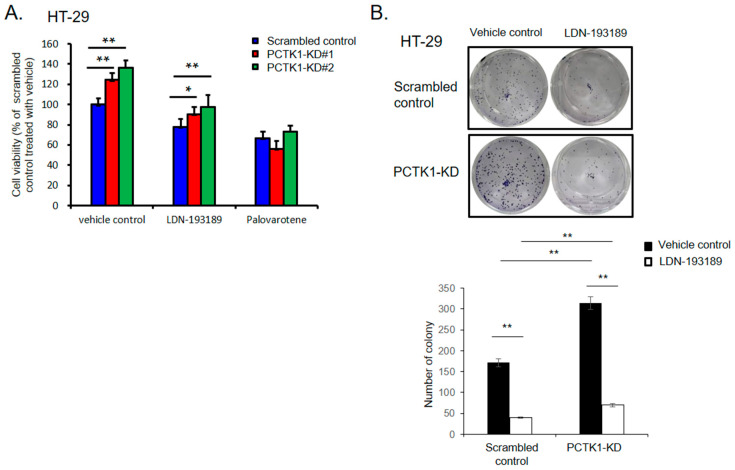

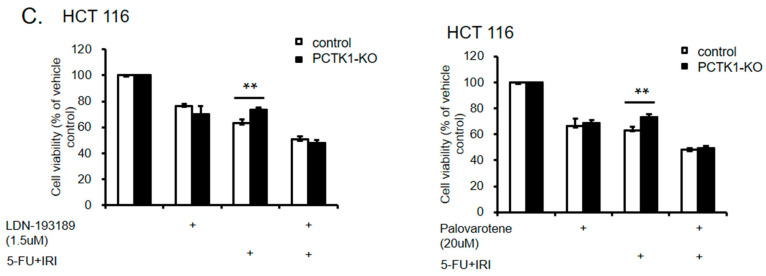
Smad1/5/8 inhibitors reversed the effect of PCTK1-KO on the promotion of malignancy and chemoresistance. (**A**) Cell proliferation of scrambled control and PCTK1-KD HT-29 cells was determined using the SRB assay after Smad1/5/8 inhibitor, LDN193189, and palovarotene treatment. The viability of the scrambled vehicle-treated control cells was set as 100%. (**B**) Representative image of colony formation in response to vehicle or LDN193189 treatment in scrambled control and PCTK1-KD HT-29 cells. (**C**) Cell viability was measured using the SRB assay in control and PCTK1-KO HCT116 cells treated with chemotherapeutics in the presence or absence of Smad1/5/8 inhibitors. * *p* < 0.05, ** *p* < 0.01.

To further evaluate the regulation of PCTK1 in cancer cell phenotype and chemoresponse through BMPR1B-SMAD1/5/8 in CRC, we treated PCTK1-KD HT-29 cells with two SMAD1/5/8 inhibitors, LDN193189 and palovarotene. As the data showed, both of these inhibitors suppressed cell proliferation and colony formation in CRC cells. However, PCTK1 KD cells were more sensitive to SMAD1/5/8 inhibitors. The difference in proliferation between control and PCTK1 KD cells was reversed in the presence of SMAD1/5/8 inhibitors for 48 h and the reduction in colony formation was also higher in cells after SMAD1/5/8 inhibitor treatment (Figure 7A,B). Moreover, both LDN193189 and palovarotene also effectively reversed the chemoresistance of PCTK1-KO cells to IRI–5-FU treatment (Figure 7C). Altogether, our data indicated that PCTK1 suppressed cell proliferation, CSC properties, and chemoresponse in CRC through the BMPR1B–-SMAD1/5/8 signaling pathway. Targeting the BMPR1B–SMAD1/5/8 signaling by pharmacological inhibition reversed the promotion of PCTK1 knockout on the CRC cell malignant phenotype and chemoresistance.

## 3. Discussion

PCTK1 is associated with diverse biological functions, including neurite outgrowth, secretory transport, and insulin secretion [30]. Teruki et al. demonstrated that loss of PCTK1 suppressed the proliferation of melanoma, prostate, breast, and cervical cancer cells [9,10]. Wang et al. reported that PCTK1 is overexpressed in lung cancer and plays an essential role in cancer cell growth and anti-apoptosis [31]. A high level of PCTK1 in the plasma correlates with poor progression-free survival in non-small-cell lung cancer [32]. Moreover, PCTK1 is involved in regulating apoptosis and disease progression in various cancers. An in vivo study of CRC and melanoma demonstrated that PCTAIRE1 siRNA–lipid nanoparticles successfully reduced tumor volume and triggered apoptosis [33].

BMPR1A and BMPR1B are both type 1 BMP receptors, which belong to a family of transmembrane serine/threonine kinases. BMPs bind BMPR1A to induce osteogenic signaling and various cellular functions through Smad-mediated signaling. BMPR1B transcription was found to be regulated by Smad via a feedback mechanism [34]. In another study, BMP receptors were observed to be involved in regulating disease progression and chemoresponse in various cancers. Pickup et al. suggested that BMPR1A acts as a tumor promoter [35]. Knockout of BMPR1A in a mouse model of mammary tumors delayed tumor initiation and prolonged survival in human breast cancer [35]. However, a reduction in BMPR1B expression induced tumor proliferation in breast cancer [36]. Jeanpierre et al. revealed that higher levels of BMPR1B in leukemic stem cells contributed to poor response to chemotherapy [37]. Furthermore, promoting leukemic cell cycle re-entry and differentiation by targeting the BMPR1B and Jak2 pathways reduced the quiescence of leukemic stem cells [37]. Dai et al. reported that low BMPR1B expression in breast cancer was associated with poor overall survival and resistance to taxanes and anthracyclines [38]. A study indicated that BMPR1B, Stat3, and BMP4-niche signaling pathways regulate leukemia remission [37]. When dimeric BMP4 binds to the BMPR receptor complex, BMPR2 phosphorylates BMPRI in their intracellular kinase domain. Moreover, receptor-specific Smad1/5/8 is recruited to the receptor complex and phosphorylated. Phosphorylated Smad1/5/8 then binds to Smad 4 to transmit signals to the nucleus [34]. Tomohiko et al. revealed that the phosphorylation of Smad1/5/8 (a downstream mediator of BMP signaling) and the expression of the downstream gene *ID3* were induced by BMP signaling overexpression and suppressed by BMP signaling KD in ovarian cancer [39]. Similarly, our data also determined that loss of PCTK1 upregulates BMPR1B signaling. 

IRI combined with 5-FU is the standard first-line chemotherapy for advanced CRC. Whether used alone or in combination with 5-FU, IRI is an adjuvant or palliative treatment for mCRC [40]. Moreover, IRI combined with continual leucovorin/5-FU infusion was superior and less toxic than was a combination of leucovorin/5-FU bolus regimens [41]. However, deaths following CRC recurrence due to chemoresistance remain an obstacle in clinical practice. CSCs are one of the main causes of chemoresistance in CRC, are involved in 5FU-based chemoresistance, and are responsible for cancer relapse [42]. We found that knockdown of PCTK1 led to chemoresistance against IRI as well as IRI–5-FU combination therapy.

Dysregulation of TGFβ signaling, another mechanism associated with chemoresistance, is implicated in the progression of various cancers, including lung [43], prostate [44], colon [45], breast and pancreatic cancer [46]. TGF-β inhibits the G1/S phase transition and terminates the cell cycle [47]. Present in multiple cell types, TGF-β is partly regulated by the Smad signaling pathway [46,48]. Dysregulation of Smad signaling can result in TGF-β resistance, leading to uncontrolled cell growth. LDN193189 and palovarotene are Smad1/5/8 inhibitors that have attracted scholarly attention in recent years [49,50]. LDN193189 has demonstrated favorable treatment efficacy in cell line experiments on melanoma [51], breast cancer [52], gastric cancer [53], and endometrial cancer [54]. It also inhibits tumorigenesis and the immune escape ability of tumor-initiating cells in the liver [55]. Furthermore, LDN193189 inhibited the progression of diffuse intrinsic pontine glioma in a mouse model [56]. Ongoing phase III clinical trials indicate that palovarotene (sold under the brand name Sohonos) is safe and effective. These data support palovarotene as a treatment for heterotopic ossification and fibrodysplasia ossificans progressiva [57,58].

**Figure 8:**
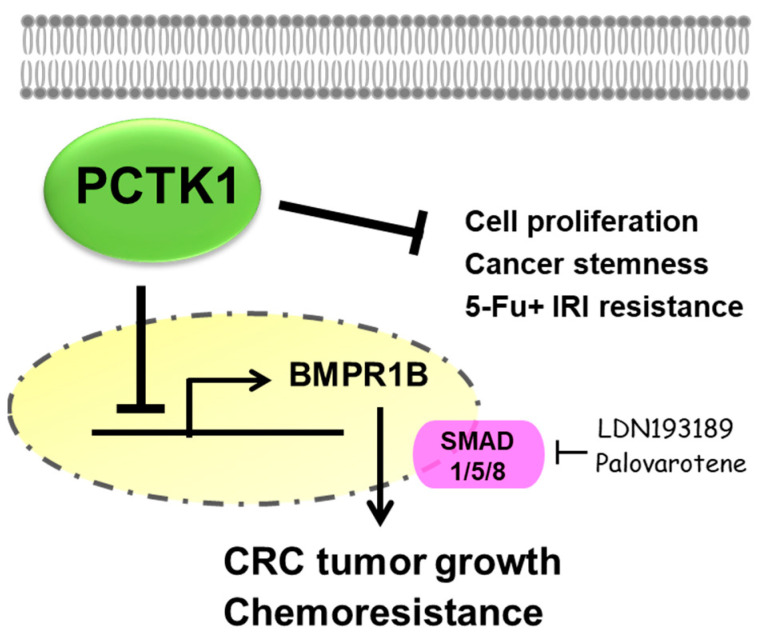
Schematic of the role of and mechanism by which PCTK1 regulates colorectal malignancy and chemoresponse. Loss-of-PCTK1 upregulates BMPR1B, one of the major components of the BMP/Smad pathway, and subsequently upregulates the downstream nuclear Smad1/5/8, which leads to CRC progression and chemoresistance. Smad1/5/8 works in a positive feedback loop to activate BMPR1B. Thus, pharmacological inhibition of the BMPR1B–Smad1/5/8 pathway by either LDN193189 or palovarotene inhibits the loss-of-PCTK1-mediated CRC tumor growth and chemoresistance.

BMP receptors have been reported to regulate cancer progression, and they are known to be regulated by Smad transcription factors. Interestingly, we found a negative association between PCTK1 and BMPR1B from the online datasets, which led us to investigate whether PCTK1 negatively regulates BMPR1B. We found that silencing BMPR1B reversed the enhancement of cell proliferation, CSC properties, and chemoresistance in PCTK1-KO cells. Treatment with Smad1/5/8 inhibitors, LDN193189, and palovarotene reduced cancer malignancy and chemoresistance in PCTK1-KD and PCTK1-KO cells. In sum, our results indicate that PCTK1 suppresses cancer progression and improves chemoresponse by downregulating the BMPR1B–Smad1/5/8 signaling pathway in CRC (Figure 8).

## 4. Materials and Methods

### 4.1. Transfection and Generation of Stable Clones

All the cell lines used in this study (DLD-1, HCT 116, and HT-29) were purchased from American Type Culture Collection (ATCC) and maintained in RPMI with 10% fetal bovine serum (FBS) supplemented with 1% penicillin-streptomycin. Cells were sub-cultured twice a week and incubated in a humidified chamber (37 °C, 5% CO_2_). Short hairpin RNA (shRNA) targeting human PCTK1 was purchased from the RNAi Core Facility at Academia Sinica, Taipei, Taiwan. Nontarget shRNA and PCTK1-shRNA were transfected into CRC cells, and puromycin was employed to select stably transfected cells for 2 weeks. Western blotting and reverse transcription quantitative real-time polymerase chain reaction (RT-qPCR) were conducted for PCTK1 level determination. pENTER-CMV-CDK16v1 (CAT#: CH889280, ViGene Biosciences, Rockville, MD, USA) was transfected into CRC cells to overexpress PCTK1 through electroporation and treatment with G418 to obtain stably transfected cells. After the overexpression pattern was confirmed through RT-qPCR and Western blot analysis, the cells were used for subsequent experiments.

### 4.2. Generation of PCTK1 Knockout Cell Lines by Using the CRISPR/Cas9 Technology

PCTK1 knockout (KO) HCT116 and DLD-1 cells were generated using the CRISPR/Cas9 technology. Two sgRNAs targeting the second and sixth coding exons were cloned separately into pAll-Cas9. The pSuperior plasmid was obtained from the National RNAi Core Facility (Academia Sinica, Taipei, Taiwan). The following targeting sites were used: 5′-GGCGATCTGAGCAAGGGACAAGG-3′ for sgRNA#1 and 5′-GTCATGTAGCGTAACGATGTTGG-3′ for sgRNA#2. Two sgRNA plasmids were transfected into HCT116 cells by using the Lipofectamine 3000 transfection reagent (Thermo Fisher Scientific Inc., Waltham, MA, USA). After 2 days, transfected cells were selected with 2 μg/mL puromycin for 1 week. Viable cells were diluted in a 96-well plate for the isolation of single-cell clones. PCTK1-KO cells were confirmed through Western blot analysis and DNA sequencing of genomic regions (Figure S1).

### 4.3. Cell Proliferation/Viability

Cells were incubated in a 5% CO_2_ humidified incubator at 37 °C for specific time periods after being seeded into a 96-well plate at a density of 5 × 10^3^ cells/well or exposed to chemotherapeutic drugs for 48 h. The cells were fixed with 10% trichloroacetic acid at 4 °C overnight, and protein-bound sulforhodamine B (SRB; 0.4% *w*/*v*) was used to stain the cells for 30 min at room temperature. Stained cells were washed twice with 1% acetic acid. Protein-bound dye was solubilized in 10 mM Tris-base solution after air drying overnight, and the OD was measured at 515 nm using a microplate reader (Bio-Rad Laboratories, Hercules, CA, USA).

### 4.4. Evaluation of Cell Proliferation Using the x-CELLigence Biosensor System

The xCELLigence Real-Time Cell Analysis (RTCA) Dual-Purpose instrument (ACEA Biosciences, Inc., San Diego, CA, USA) was employed to analyze cell proliferation and migration as previously described [59]. The cell growth rate was determined using an E-plate 16 (ACEA Biosciences, Inc.). Cells were monitored once every 30 s for 4 h. After being seeded on an E-plate in FCS-containing medium at a density of 5000 cells per well, they were monitored every 30 min. The data were analyzed using RTCA software 1.2 (supplied with the instrument).

### 4.5. Colony Formation Assay

The cells were cultured in an incubator containing 5% CO_2_ at 37 °C after being seeded in 6-well plates at a density of 1 × 10^3^ cells/well or exposed to chemotherapeutic drugs. Subsequently, cells were fixed with 0.4% formaldehyde and stained with 0.1% crystal violet for 10 days. Colony number was determined using Image J software (version 1.53) or a hand counter.

### 4.6. Sphere Formation Assay

Cells in stem cell medium consisting of serum-free RPMI 1640 medium supplemented with 10 ng/mL human basic fibroblast growth factor (bFGF; Invitrogen, Grand Island, NY, USA), 20 ng/mL epidermal growth factor (Invitrogen), and 1× B27 supplement were seeded in ultralow-attachment six-well plates (Corning, Corning, NY, USA). The spheroids formed were counted and photographed after 14 days of incubation.

### 4.7. In Vivo Tumor Xenograft Experiments

We established DLD-1 cells with the stable integration of scrambled control and PCTK1 overexpression (PCTK1-over) as well as PCTK1-KO. Five-week-old male Nu/Nu mice were used as the in vivo experimental model. The flank of each mouse was subcutaneously injected with 0.1 mL (concentration: 10^7^ cells/mL) of PCTK1-over, vector control, scrambled control, or PCTK1-KO cells. Tumor dimensions and body weights were measured twice a week. Subcutaneous tumors were measured using the following equation: (L × w2)/2. After the mice were killed, tumors were excised and weighed. For RNA and protein analysis, the xenografts were flash-frozen on dry ice and stored at −80 °C. All animal use protocols were approved by the Institutional Animal Care and Use Committee of Taipei Medical University (LAC-2014-0401).

### 4.8. Annexin V/Propidium Iodide Double Staining Assay

Following 48 h chemotherapeutic treatment, cells were detached and washed twice with ice-cold PBS. Subsequently, cell pellets were resuspended in staining buffer (annexin V–FITC, propidium iodide solution, and annexin V binding buffer; Strong Biotech Corporation, Taipei, Taiwan) and further incubated in the dark at room temperature for 15 min. Annexin V binding buffer was added to the cell suspension, after which flow cytometry (BD FACSVerse Cell Analyzer, BD Biosciences) was conducted.

### 4.9. RT-qPCR

RNAzol was used to extract total RNA from the samples according to the manufacturer’s protocol (Molecular Research Center, Cincinnati, Inc., Cincinnati, OH, USA). The ABI kit was used for reverse transcription of total RNA (Applied Biosystems, Waltham, MA, USA). With GADPH as the internal control, RT-qPCR of PCTK1, NANOG, SOX2, Oct4, CD44, CD133, and EpCAM was performed with 200 ng of complementary DNA and 0.5 μM of forward and reverse primers of PCTK1 (Forward: GCAGTGACCCTGGAGAGG, Reverse: TCAAGTCCTCGTGCACAATC), NANOG: Forward: CCAAAGGCAAACAACCCACTT, Reverse: CGGGACCTTGTCTTCCTTTTT, SOX2: Forward: ACAGCAAATGACAGCTGCAAA, Reverse: TCGGCATCGCGGTTTTT, Oct4: Forward: CGACCATCTGCCGCTTTG, Reverse: GGGCCGCAGCTTACACAT, CD44: Forward: CAGATGGCATGAGGGATATCG, Reverse: CTGCAGCTGTCCCTGTTGTC, CD133: Forward: ATCTGCAGTGGATCGAGTTCTCT, Reverse: GCGGTGGCCACAGGTTT, EpCAM: Forward: TTATGATCCTGACTGCGATGAGA, Reverse: GGTGCCGTTGCACTGCTT, and GAPDH: Forward: CCTGTACGCCAACACAGTGC, Reverse: ATACTCCTGCTTGCTGATCC) and 2× SYBR Green Master Mix (Bio-Rad Laboratories, Hercules, CA, USA) in a final volume of 10 μL. Reverse transcription polymerase chain reaction (RT-PCR) was performed in triplicate for each sample.

### 4.10. Western Blotting

Cells were suspended in lysis buffer (Sigma-C2978) with a protease inhibitor cocktail (SLBK4607V, Sigma-Aldrich, Saint Louis, MI, USA) to obtain protein lysates. The cell suspension was centrifuged at 13,000 rpm for 15 min to extract the supernatant. The Bio-Rad Protein Assay Kit (Bio-Rad Laboratories, Inc., Hercules, CA, USA) was used to measure protein concentrations (μg/mL) by using a Bio-Rad Model 680 microplate reader at a wavelength of 595 nm. For cytosolic and nuclear protein fractionation, we followed the protocol as described previously [60]. Next, acrylamide gel electrophoresis (with 10–15% acrylamide) was performed using aliquots of cell lysates containing 20 μg of total protein and then transferred to polyvinylidene fluoride membranes (Pall Corp, Prot Washington, NY, USA). The membranes were probed overnight at 4 °C with primary antibodies, namely, GAPDH (IR3-8, iReal Biotechnology Inc., Hsinchu, Taiwan), PCTK1 (HPA001366, Atlas Antibodies, Bromma, Sweeden), c-PARP (IR101–420, iReal Biotechnology Inc., Hsinchu, Taiwan), Bcl2 (2872S, Cell Signaling Technology), Bcl-xL (AHP1722, Bio-Rad), Bax (IR93–389, iReal Biotechnology Inc., Hsinchu, Taiwan), p53 (sc-393031, Santa Cruz Biotechnology, Dallas, TX, USA), c-caspase 3 (IR96–401, iReal Biotechnology Inc., Hsinchu, Taiwan), Smad1/5/8 (sc-6031-R, Santa Cruz Biotechnology). Horseradish-peroxidase-conjugated donkey anti-mouse and anti-rabbit secondary antibodies (Santa Cruz Biotechnology, Dallas, TX, USA) were used to detect the primary antibodies. Visualization was achieved with the Versa Doc Imaging system by using the SuperSignal West Pico Chemiluminescent Substrate (Amersham Biosciences, Amersham, UK).

### 4.11. Bioinformatics Data Resources

The GEO database and Kaplan–Meier plots were constructed. For the quantitative evaluation of overall survival, chi-square results were used to understand the relationship between PCTK-1 gene expression and CRC survival. The role of PCTK1 in survival in CRC was assessed using the Gene Expression Omnibus (GEO) (http://www.ncbi.nlm.nih.gov/geo/) [61] dataset GSE41258 that contained gene expression data and clinical information of patients with CRC. The total number of cases were 252. The z-scores were calculated in the dataset to identify if the expression of the CDK16 gene. Low expression was a negative z-score and a positive z-score was high expression. The z-score was calculated by the formula z = (x − μ)/σ, where x is gene expression value in a specific patient, μ is the average gene expression, and σ is the standard deviation of the datasets gene expression. The overall survival (OS) in months was assessed by comparing the survival profiles on the basis of a high or low expression of the gene. These were presented by Kaplan–Meier survival curve plots using SPSS for Macintosh (version 21; IBM Corp., Armonk, NY, USA; www-01.ibm.com), and log-rank *p* value (<0.05 as significance value) was calculated.

A clinical evaluation of PCTK1 was conducted using the R2: Genomics Analysis and Visualization Platform (http://r2.amc.nl). Kaplan–Meier survival curves were generated using the “Mixed Tumor Colon—Medema—108—MAS5.0—u133p2” dataset (GSE33114) for relapse-free survival (RFS) and the “Tumor Colon—Smith—232—MAS5.0—u133p2” dataset (GSE17538) for overall survival (OS). The “curtain” mode was employed to determine the threshold point, and a significance level of *p* < 0.05 was applied.

GO and KEGG enrichment analysis was performed using the clusterProfiler package [4.4.4]. The visualizations were generated in R using the ggplot2 graphics package.

The ROC Plotter, a web-based transcriptome analysis tool for validating predictive biomarkers of therapy response (https://www.rocplot.org/), was employed to evaluate the predictive potential of genes with chemotherapy response.

The GEPIA dataset (GSE database) was used to clinically validate the DEGs involved in the aforementioned pathways. The Oncomine tool (https://www.oncomine.org/resource/login.html) was used to represent the average expression levels of all target genes in normal versus tumor tissues. Independent comparisons of the expression levels of these genes were made to overall survival in months obtained using GEPIA (http://gepia.cancer-pku.cn/) and oncogenomics web tools (https://hgserver1.amc.nl/cgi-bin/r2/main.cgi). Kaplan–Meier survival curves were plotted. Hazard ratios with 95% confidence intervals and log-rank *p* values were calculated. The level of significance was set at *p* < 0.05.

### 4.12. Gene Set Enrichment Analysis

We used a *p* value of <0.05, a fold change value of ≥2, and a false discovery rate of <0.05 as the screening criteria in the gene set enrichment analysis (GSEA).

### 4.13. cBioPortal

We used the open access resource cBioPortal (http://www.cbioportal.org/) to search for multidimensional cancer genomic datasets. The co-expression of PCTK1 and BMPR1B was analyzed using cBioPortal.

### 4.14. Statistical Analysis

Data from at least three independent experiments are presented as means ± standard deviations. The two-tailed Student’s *t* test was performed to detect significant differences between two groups of data, with *p* < 0.05 considered significant.

## 5. Conclusions

We discovered that PCTK1 acts as a tumor suppressor in CRC using bioinformatics, a cell model, and a xenograft mouse model. We determined the negative regulatory role of PCTK1 on the proliferation and cancer stemness features of CRC cells by overexpression, KD, and CRISPR/Cas9 KO models. Additionally, decreased PCTK1 contributed to resistance in both single-agent and combination chemotherapy (i.e., IRI and IRI–5-FU). Furthermore, PCTK1-KO cells’ malignance and chemoresistance were restored by inhibiting BMPR1B and Smad1/5/8. Altogether, our study points to the possible utilization the PCTK1/BMPR1B–Smad1/5/8 axis as a therapeutic target for CRC. Inclusion of Smad1/5/8 inhibitors in the therapeutic regimen might also curb the chemoresistance in CRC patients with low PCTK1 levels.

## Data Availability

The dataset supporting the conclusions of this article is included within the article.

## Supplementary Materials

The supporting information can be downloaded at: https://www.mdpi.com/article/10.3390/ijms241210008/s1.

## Abbreviations

PCTK1: PCTAIRE protein kinase 1; CRC: colorectal cancer; BMP: bone morphogenetic protein.
