# Supplementary material for: PCTAIRE Protein Kinase 1 (PCTK1) Suppresses Proliferation, Stemness, and Chemoresistance in Colorectal Cancer through the BMPR1B-Smad1/5/8 Signaling Pathway
Source: Int J Mol Sci. 2023 Jun 11;24(12):10008. doi: 10.3390/ijms241210008 (PMC10298136; doi:10.3390/ijms241210008)
Supplement: Supplementary file 1 [file ijms-24-10008-s001.zip › ijms-2420581-supplementary.pdf]

A

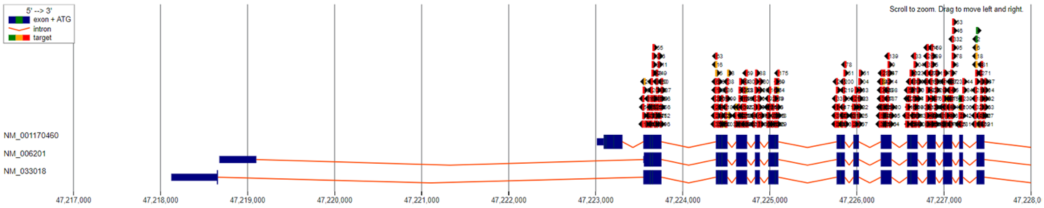

B

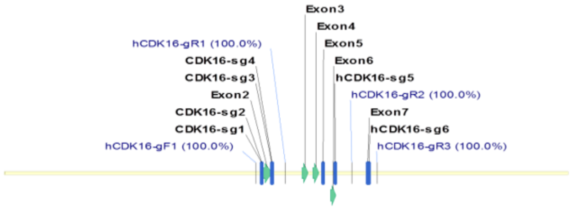

PCTK1-sg1:GGCGATCTGAGCAAGGGACA AGG

PCTK1-sg6:GTCATGTAGCGTAACGATGT TGG

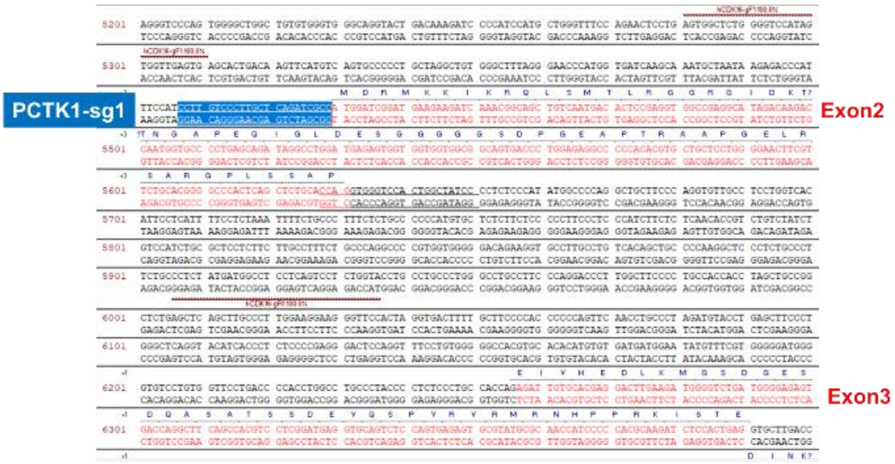

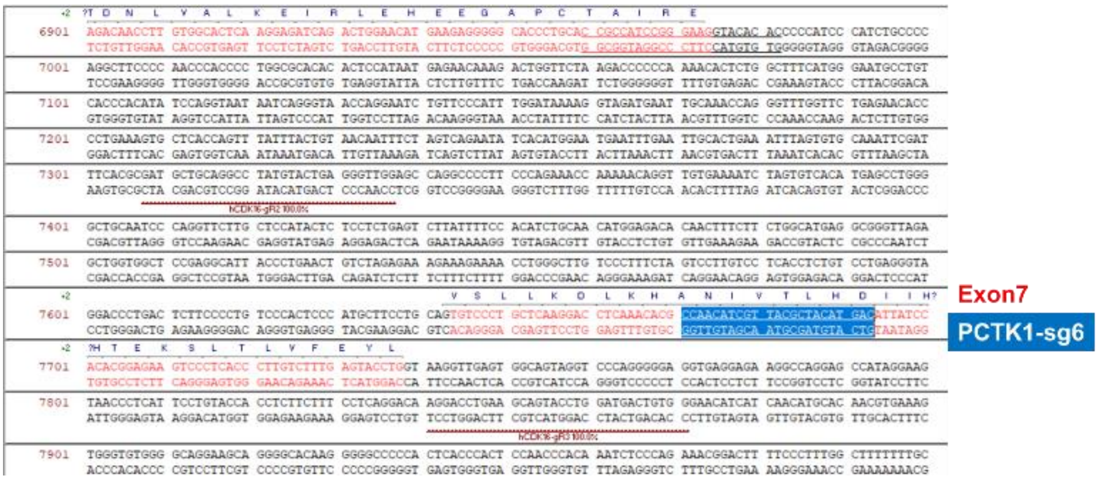

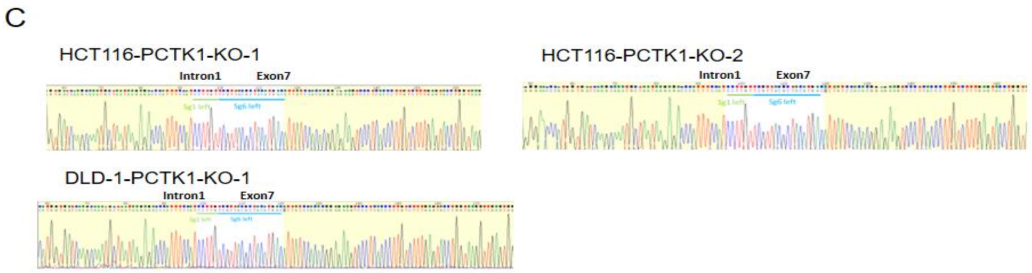

Supplementary Figure S1. Generation of PCTK1 Knockout CRC cell lines by using the CRISPR/Cas9 technology.

A) Design guide RNA (sgRNA) for PCTK1 PCTK1-coding region (the Gene ID: 5127, https://chopchop.cbu.uib.no/). B) Two sgRNAs (sg1/ sg6) target the 2nd and 6th coding exon were separately cloned into pAll-Cas9. C). The PCTK1 knockout cells (HCT 116 and DLD-1) were confirmed by DNA sequencing of the genomic regions.

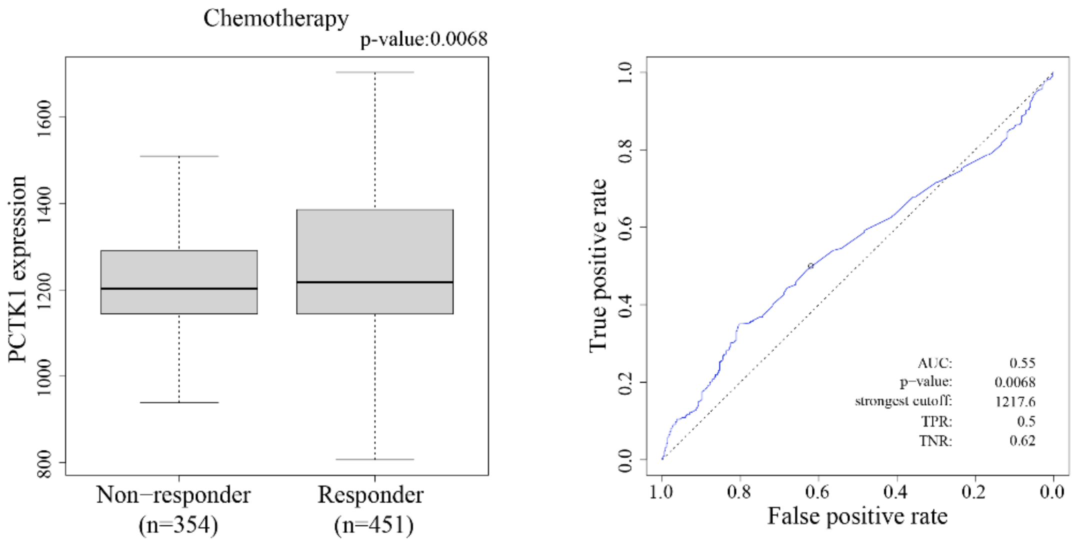

Supplementary Figure S2 Box plot was employed to depict the expression of PCTK1 in colorectal cancer patients categorized based on their chemotherapy responses using the Response Evaluation Criteria in Solid Tumors (RECIST) criteria. Receiver operating characteristic (ROC) analysis was performed to evaluate the prognostic significance of PCTK1 expression in overall survival. The area under the curve (AUC), true positive rate (TPR), and true negative rate (TNR) were calculated as performance metrics.

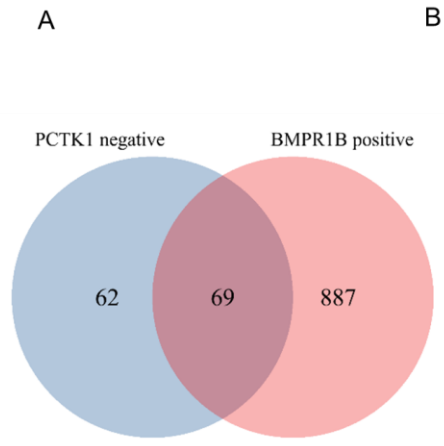

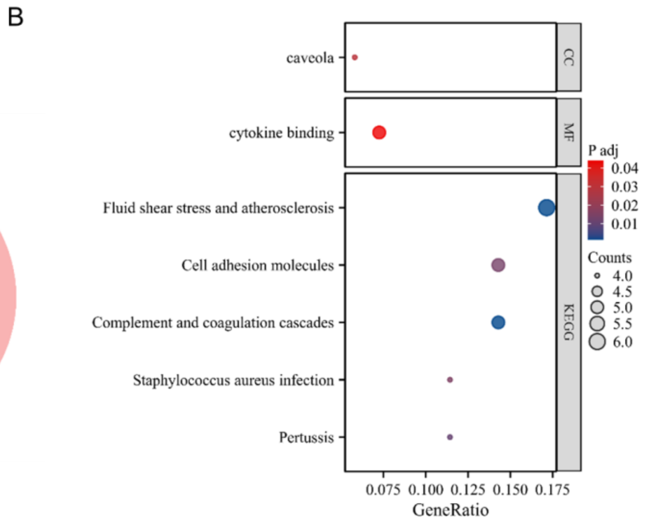

Supplementary Figure S3 GO/KEGG analysis of PCTK1 and BMPR1B in colorectal cancer. (A) The Venn diagram illustrates the overlap between the PCTK1 negatively correlated co-expressed genes and the BMPR1B positively correlated co-expressed genes. (B) The results of GO/KEGG pathways enriched from commonly co-expressed PCTK1 and BMPR1B genes in colorectal cancer.

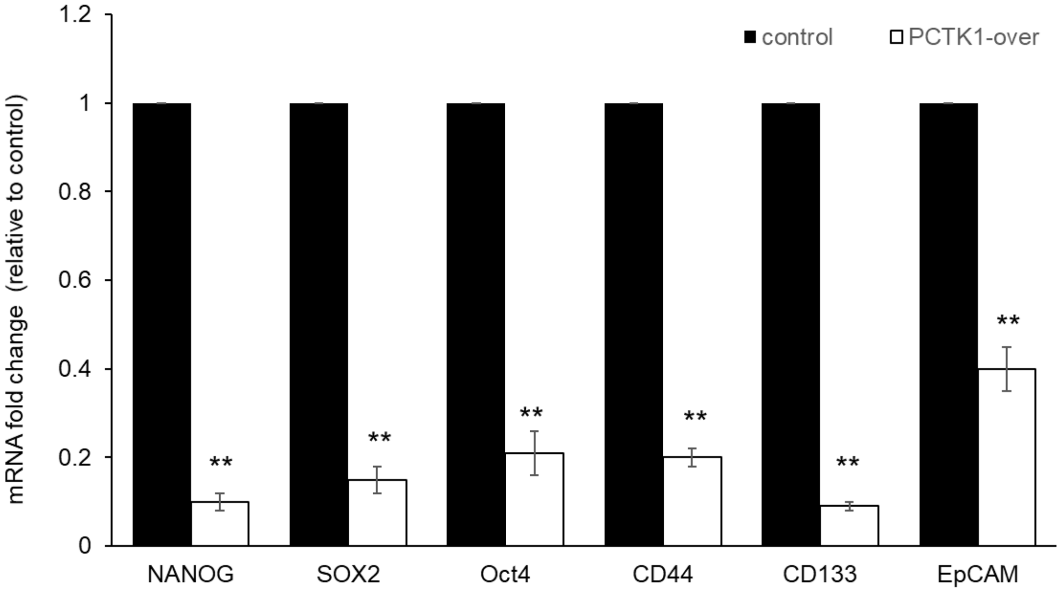

Supplementary Figure S4 mRNA levels of NANOG, SOX2, Oct-4, CD44, CD133, and EpCAM in the control and PCTK1-over cells were determined through qPCR. ** Stands for p value <0.01
